# Impact of mutations in homologous recombination repair genes on treatment outcomes for metastatic castration resistant prostate cancer

**DOI:** 10.1371/journal.pone.0239686

**Published:** 2020-09-30

**Authors:** Alexander S. Carlson, Rigo I. Acevedo, Daniel M. Lim, Roman Gulati, Agnes Gawne, Alexandra O. Sokolova, Heather H. Cheng, Peter S. Nelson, R. Bruce Montgomery, Evan Y. Yu, Michael T. Schweizer

**Affiliations:** 1 University of Washington, Seattle, WA, United States of America; 2 Division of Public Health Sciences, Fred Hutchinson Cancer Research Center, Seattle, WA, United States of America; 3 Division of Oncology, University of Washington, Seattle, WA, United States of America; 4 Division of Clinical Research, Fred Hutchinson Cancer Research Center, Seattle, WA, United States of America; 5 Division of Human Biology, Fred Hutchinson Cancer Research Center, Seattle, WA, United States of America; CNR, ITALY

## Abstract

**Introduction:**

A significant proportion of patients with metastatic castration-resistant prostate cancer (mCRPC) harbor mutations in homologous recombination (HR) repair genes, with some of these mutations associating with increased tumor susceptibility to poly(ADP-ribose) polymerase (PARP) inhibitors and platinum-based chemotherapy. While mutations in some HR repair genes (e.g., *BRCA1/2*) have been associated with a more aggressive clinical course, prior studies correlating HR mutational status with treatment response to androgen receptor (AR) signaling inhibitors (ARSIs) or taxane-based chemotherapy have yielded conflicting results.

**Methods:**

We conducted a single-center retrospective analysis to assess clinical outcomes to conventional, regulatory-approved therapies in mCRPC patients with somatic (monoallelic and biallelic) and/or germline HR repair mutations compared to patients without alterations as determined by clinical-grade next-generation sequencing assays. The primary endpoint was PSA30/PSA50 response, defined as ≥30%/≥50% prostate-specific antigen (PSA) reduction from baseline. Secondary endpoints of PSA progression-free survival (pPFS) and clinical/radiographic progression-free survival (crPFS) were estimated using Kaplan-Meier methods.

**Results:**

A total of 90 consecutively selected patients were included in this analysis, of which 33 (37%) were identified to have HR repair gene mutations. Age, race, Gleason score, prior surgery, and receipt of prior radiation therapy were comparable between carriers and non-carriers. There was no evidence that PSA30/PSA50 differed by HR gene mutational status. Median pPFS and crPFS ranged 3–14 months across treatment modalities, but there was no evidence either differed by HR gene mutational status (all p>0.05). There was also no difference in outcomes between those with *BRCA2* or *PALB2* mutations (n = 17) compared to those without HR repair mutations.

**Conclusion:**

HR gene mutational status was associated with comparable clinical outcomes following treatment with ARSIs or taxane-based chemotherapy. Additional prospective studies are needed to confirm these findings.

## Introduction

DNA damage repair (DDR) pathways play an essential role in maintaining genomic integrity. Individuals harboring germline mutations in DDR genes are more susceptible to cancer development [1]. Germline and somatic DDR mutations are also associated with a more aggressive clinical course in certain cancers: germline *BRCA* mutations have been associated with decreased breast cancer-specific survival [2] and linked to poor outcomes in patients with prostate cancer, including decreased metastasis-free survival in those initially presenting with localized disease [3, 4]. The significance of DDR mutations in prostate cancer has expanded in recent years as studies have demonstrated that their prevalence is higher than previously thought [5, 6].

Importantly, DDR genes specifically involved in homologous recombination (HR) repair may be predictive for response to poly(ADP-ribose) polymerase (PARP) inhibitors in patients with metastatic castration-resistant prostate cancer (mCRPC). Treatment with PARP inhibitors leads to persistent single-strand DNA (ssDNA) breaks through inhibition of base excision repair. These ssDNA breaks then degenerate to double-strand DNA (dsDNA) breaks and, because HR repair deficient cells are unable to efficiently repair dsDNA breaks, PARP inhibitors are synthetically lethal to these cancer cells [7, 8]. Various other mechanisms likely contribute to PARP inhibitor sensitivity in HR repair deficient tumors, including PARP1 trapping and creation of cytotoxic PARP1-DNA complexes at sites of endogenous damage, the promotion of nonhomologous end joining (NHEJ) activity, and inhibition of DNA repair protein recruitment (e.g., *BRCA1*, *BARD1*) [9, 10].

The two-stage TOPARP trials revealed high response rates to olaparib in mCRPC patients with HR repair gene mutations who were no longer responding to standard therapies [11, 12]. More recently, the phase III PROfound trial demonstrated increased radiographic progression-free survival (PFS) and objective responses in mCRPC patients with HR repair gene mutations receiving olaparib compared to enzalutamide or abiraterone [13], although activity in the patients with mutations in non-*BRCA*-mutated HR repair genes remains uncertain [12, 14, 15]. The phase II TRITON2 study found no clear evidence of response to rucaparib in patients with *ATM*, *CDK12*, and *CHEK2* mutations, whereas patients with mutations in genes that directly interact with the *BRCA* complex (e.g. *PALB2*, *FANCA*, *RAD51*, etc.) showed promising radiographic and prostate-specific antigen (PSA) response [15]. Rupacarib has since gained accelerated FDA approval for mCRPC patients with deleterious *BRCA1/2* mutations who were previously treated with novel androgen receptor (AR) targeted therapy and taxane chemotherapy [16]. Olaparib has received full approval for mCRPC patients who have at least one line of novel AR targeted therapy and have a suspected or known deleterious HR repair mutation across a broad panel [17].

The presence of HR repair gene mutations in cancers has also been associated with enhanced sensitivity to platinum-based chemotherapy. The likely mechanism behind this sensitivity is through the formation of dsDNA breaks via DNA adducts [18].

Cheng, et al. reported that patients with biallelic *BRCA2* inactivation can achieve excellent clinical response with carboplatin even after progression on first-line therapies for mCRPC [19]. Germline *BRCA2* variants were also found to strongly associate with PSA response ≥50% in mCRPC patients treated with carboplatin [20].

While significant effort has gone into exploring precision medicine approaches for treating prostate cancer patients with HR repair gene mutations, there are limited reports describing the clinical course of these patients following treatment with standard therapies. Androgen receptor signaling inhibitors (ARSIs), such as abiraterone and enzalutamide, as well as taxane-based chemotherapy, such as docetaxel and cabazitaxel, have been established as standard, regulatory-approved treatment options for patients with mCRPC [21]. Recently, Hussain, et al. reported that somatic HR repair mutations may be associated with improved PSA response and PFS with abiraterone [22]. This contrasted with previous findings of attenuated ARSI response in germline HR gene mutation carriers [23]. Studies examining taxane-based chemotherapy treatment in mCRPC found no significant difference in treatment responses when stratifying by HR gene mutation status [24, 25]. Notably, treatment with cabazitaxel was not evaluated in these reports.

The objective of this paper is to investigate how mutations in genes directly and indirectly involved in the HR repair pathway impact treatment response and long-term outcomes in mCRPC patients treated with abiraterone, enzalutamide, docetaxel, and cabazitaxel. We hypothesized that the presence of HR repair gene mutations will correlate with poor clinical outcomes following treatment with these agents.

## Materials and methods

### Patients

A retrospective analysis was performed using the institutional Caisis database, which includes prostate cancer patients treated at the University of Washington/Seattle Cancer Care Alliance (UW/SCCA). Prior to data abstraction, this project was reviewed by the Institutional Review Board at the University of Washington and deemed to be minimal risk. As such, the requirement for informed consent was waived. Patient electronic medical records were accessed between 05/2018-02/2020 and were not anonymized to data abstractors. All patient data was de-identified prior to performing statistical analysis.

Our inclusion criteria mandated that patients have pathologically proven prostate cancer and documented mCRPC status, defined as disease progression following surgical/medical castration (i.e. androgen deprivation therapy; ADT) by PSA or radiographic/clinical evidence. Additionally, patients must have previously undergone clinical-grade next-generation sequencing (NGS) of their tumor. An exception was made to include patients with known germline *BRCA2* mutations who did not undergo tumor sequencing, given that these germline cases are most often associated with the loss of the second allele by somatic mutation [6]. The following assays were included: UW-OncoPlex, FoundationOne, Guardant360, GeneTrails, BROCA, Color, BRACAnalysis, and Whole Exome Sequencing (WEC) assay results [5].

We specifically analyzed monotherapy treatments with each of the four agents (abiraterone, enzalutamide, docetaxel, cabazitaxel) that occurred between 2011–2019. Per Prostate Cancer Working Group 3 (PCWG3) guidelines, we required a minimum 60 days of therapy in order to assess responses and progression endpoints [26]. Patients treated with more than one of the four agents were eligible for analysis in multiple treatment groups if all above inclusion criteria were met. If a patient received multiple courses of the same agent, only the first treatment course was assessed. Confirmation of PSA response/progression was not required since this was not uniformly performed in these non-trial patients. Patients with neuroendocrine or small cell differentiation were excluded unless their treatments occurred prior to pathological confirmation of neuroendocrine/small cell transdifferentiation.

### HR repair gene status

Patients were sub-divided into two groups based on the presence or absence of HR repair gene mutations. The HR group contained patients with both somatic and germline mutations in genes involved in HR repair, including those indirectly regulating this pathway [5, 6, 27]. A broad set of genes was included given the recent approval of olaparib across an inclusive set of genes both directly and indirectly involved in HR repair [13, 17]. Monoallelic somatic mutations were considered sufficient for inclusion, given that many assays (e.g., FoundationOne) do not assess for loss of heterozygosity (LOH) or explicitly report germline mutations. Mutations were considered pathogenic if reported as such on the clinical report. Variants of unknown significance or otherwise benign changes were not included in the HR repair mutation cohort.

### Data endpoints

The primary objective of this study was to determine the PSA50 and PSA30 response rate for each therapy, which was defined as the proportion of patients achieving ≥50% and ≥30% PSA reduction from the baseline PSA, respectively. Nadir PSA was recorded as the lowest PSA after starting treatment and prior to initiating a subsequent therapy.

The secondary objectives included determining PSA PFS (pPFS) and clinical/radiographic PFS (crPFS). PSA progression was defined by a PSA increase that was ≥2 ng/mL and ≥25% above the nadir on two consecutive lab draws. Clinical/radiographic progression was based on the assessment of the treating physician and was determined through chart review. Response Evaluation Criteria in Solid Tumors (RECIST) criteria were not utilized given that most patients were not treated on a clinical trial.

### Statistical analysis

Differences in demographic characteristics and baseline laboratory measurements between the two populations were evaluated using Kruskal-Wallis or Fisher’s exact tests. Differences in PSA50 and PSA30 between populations were evaluated using Fisher’s exact tests separately for each treatment modality. Kaplan-Meier curves were calculated for pPFS and crPFS for each treatment modality, and differences between populations were evaluated using log-rank tests. A p-value <0.05 was considered statistically significant. All analyses were carried out using R statistical software version 3.6.3.

A secondary analysis estimated probabilities of PSA30/PSA50 using Bayesian logistic regression to adjust for prior receipt of similar therapy. Adjustment for prior similar therapy was examined given numerous studies highlighting varying levels of cross-resistance in these treatment modalities [28–32]. Prior similar therapy was defined as prior receipt of abiraterone or enzalutamide when either ARSI treatment was analyzed and prior receipt of docetaxel or cabazitaxel when either taxane-based chemotherapy was analyzed.

An exploratory analysis compared outcomes specifically for patients with *BRCA2* and *PALB2* mutations to those without HR repair gene mutations. Previous literature has demonstrated that these genes are closely associated with HR repair functional status [33, 34] and appear to be important predictive biomarkers for determining eligibility for DNA damaging agents. PSA30/PSA50 and pPFS/crPFS outcomes were re-evaluated for these comparisons.

## Results

### Patients

A total of 90 consecutively selected patients were included in this analysis, with HR repair gene mutations identified in 33/90 (37%). Of the 20 patients who underwent dedicated germline testing, 5 (25%) were found to harbor germline HR repair alterations. Mutations were found in 8 unique HR repair genes, with *BRCA2* (n = 16) being the most frequently altered (Table 1, S1 Table). A demographic comparison based on HR status showed similarity in age at diagnosis, Gleason score, prior surgery, and receipt of prior radiation therapy between the two patient populations (Table 2). Over 21% of patients with HR repair mutations were non-white in comparison to 7.1% of patients without HR repair mutations, although this finding did not reach statistical significance (p = 0.07). Laboratory parameters assessed prior to starting each treatment were also similar between the populations (S2–S5 Tables).

**Table 1:** Complete list of HR mutations.

Study ID	Sequencing Assay	Affected HR Gene	Germline alteration?	Bi-allelic mutation?
1	UW-OncoPlex	*FANCA*	N/A	No
2	WEC	*BRCA2*	No	Yes
4	UW-OncoPlex	*BRCA2*	N/A	Yes
9	UW-OncoPlex	*MRE11A*	N/A	No
14	UW-OncoPlex	*BRCA2*	No	Yes
20	UW-OncoPlex	*BRCA2*	No	Yes
24	FoundationOne, Color	*BRCA2*	Yes	Yes
25	UW-OncoPlex	*CDK12*	N/A	Yes
27	UW-OncoPlex	*BRCA2*	N/A	No
28	UW-OncoPlex, WEC	*BRCA2*	Yes	Yes
31	UW-OncoPlex	*BRCA2*	N/A	Yes
33	UW-OncoPlex	*BRCA2*	N/A	No
34	UW-OncoPlex	*CDK12*	N/A	Yes
36	FoundationOne	*ATM*	N/A	Yes
38	UW-OncoPlex	*PALB2*	N/A	Yes
39	UW-OncoPlex	*CHD1*	N/A	No
43	UW-OncoPlex	*BRCA2*	N/A	N/A
44	Color	*BRCA2*	Yes	N/A
48	UW-OncoPlex	*CHD1*	N/A	Yes
57	FoundationOne	*CDK12*	N/A	Yes
59	UW-OncoPlex	*BRCA2*	N/A	Yes
63	BRACAnalysis	*BRCA2*	Yes	N/A
69	UW-OncoPlex	*CHD1*	N/A	Yes
70	UW-OncoPlex	*ATM*	N/A	No
71	UW-OncoPlex	*FANCA*	N/A	N/A
72	UW-OncoPlex	*CDK12*	N/A	N/A
78	Guardant360, FoundationOne, Color	*BRCA2*	Yes	N/A
79	UW-OncoPlex	*CHD1*	N/A	Yes
81	FoundationOne	*ATM*	No	No
82	GeneTrails	*FANCA*	N/A	N/A
84	UW-OncoPlex	*BRCA2*	N/A	Yes
86	UW-OncoPlex	*MRE11A*	N/A	Yes
90	UW-OncoPlex	*BRCA2*, *CHEK2*	N/A	Yes

“WEC” denotes the Whole Exome Sequencing assay.

**Table 2:** Patient characteristics by HR status.

Measure	No HR (N = 57)	HR (N = 33)	P-value
Age, years, median [IQR]	61.1 [55.2, 66.4]	61.0 [53.0, 67.5]	0.8
Race, N (%)			
• White	53 (93.0)	26 (78.8)	0.07
• Black	1 (1.8)	4 (12.1)	
• Asian/Unknown	3 (5.3)	3 (9.1)	
Gleason Score, N (%)			
• 6	2 (3.5)	0 (0.0)	0.6
• 7	20 (35.1)	10 (30.3)	
• 8–10	31 (54.4)	22 (66.7)	
• Unknown	4 (7.0)	1 (3.0)	
Prior Surgery, N (%)			
• No	31 (54.4)	20 (60.6)	0.4
• Yes	26 (45.6)	12 (36.4)	
• Unknown	0 (0.0)	1 (3.0)	
Prior Radiation Therapy, N (%)			
• No	14 (24.6)	12 (36.4)	0.6
• Yes	29 (50.9)	15 (45.5)	
• Unknown	14 (24.6)	6 (18.2)	

P-value for age from Kruskal-Wallis rank sum test and for other measures from Fisher’s exact test.

### Efficacy outcomes

**Fig 1:**
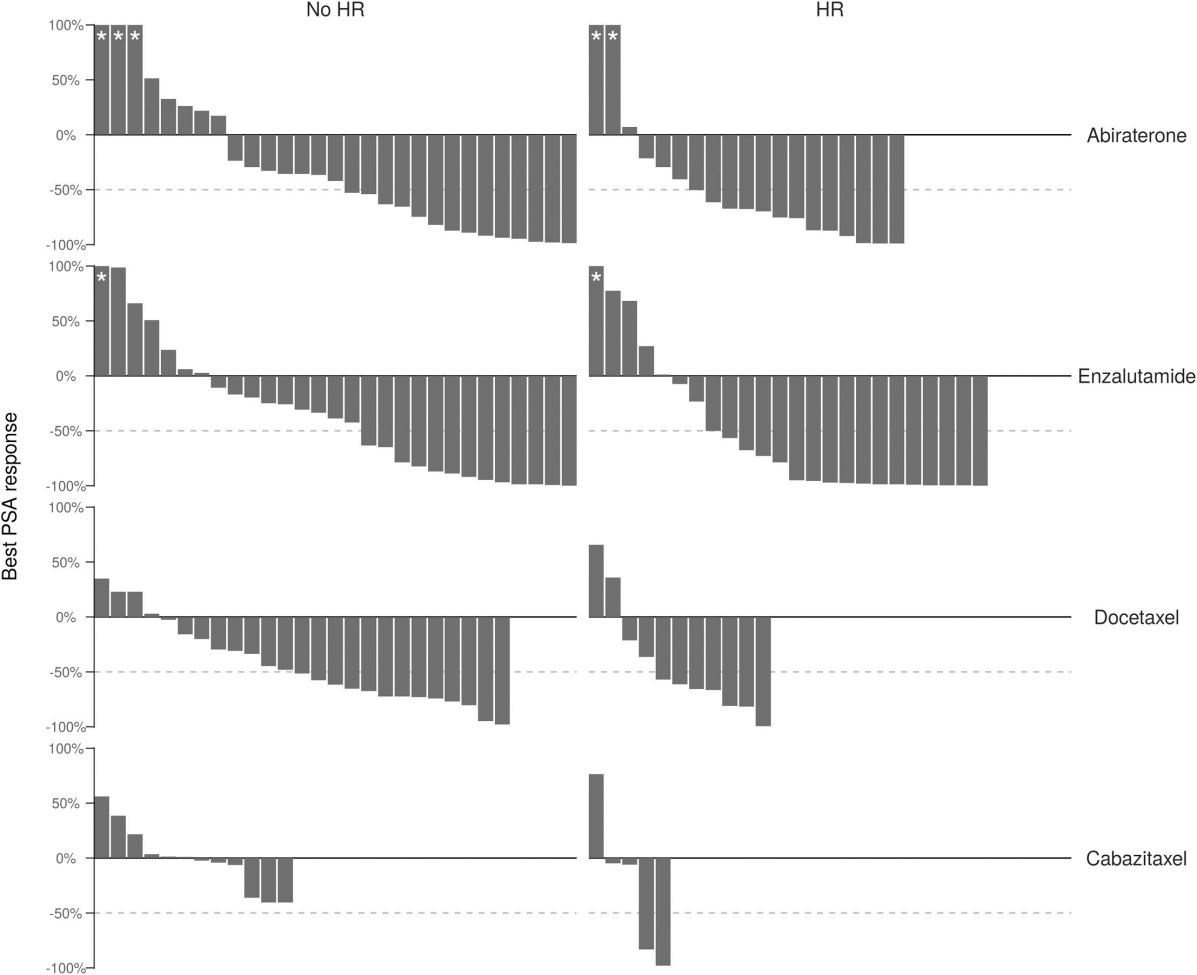
Waterfall plot of best PSA response by treatment and HR status. Maximum percent relative change from baseline during treatment or after completing treatment but prior to subsequent treatment. Maximum percent change greater than 100% is truncated at 100%. Dashed horizontal lines show 50% decrease.

Best PSA response among patients with/without HR repair gene mutations is depicted in Fig 1 using waterfall plots for each treatment modality. Most patients achieved a PSA reduction from baseline in every treatment group except for patients without HR repair mutations treated with cabazitaxel. There was no evidence that PSA30 or PSA50 differed based on HR status for any treatment modality (Table 3). The frequency of patients achieving PSA50 in the population with HR repair gene mutations was 26% higher in the enzalutamide group and 40% higher in the cabazitaxel group compared to patients with no HR repair gene mutations, though neither result was statistically significant (p = 0.09 and p = 0.07, respectively). Regardless of HR repair gene mutation status, the PSA30 response rate was >50% and PSA50 response rate was >40% for all treatment modalities except cabazitaxel. Adjusting for prior similar therapy did not reveal an association between HR repair gene mutation status and PSA30/PSA50 for any of the treatments (S6 Table).

**Table 3:** Best PSA response by treatment and HR status.

Treatment	Response	No HR, N (%)	HR, N (%)	P-value
Abiraterone	PSA30	19/29 (66%)	14/19 (74%)	0.8
	PSA50	14/29 (48%)	13/19 (68%)	0.2
Enzalutamide	PSA30	17/29 (59%)	17/24 (71%)	0.4
	PSA50	13/29 (45%)	17/24 (71%)	0.09
Docetaxel	PSA30	17/25 (68%)	8/11 (73%)	1.0
	PSA50	13/25 (52%)	7/11 (64%)	0.7
Cabazitaxel	PSA30	3/12 (25%)	2/5 (40%)	0.6
	PSA50	0/12 (0%)	2/5 (40%)	0.07

PSA50 is 50% decrease in PSA relative to baseline. PSA30 is 30% decrease in PSA relative to baseline. P-values from Fisher’s exact test.

**Fig 2:**
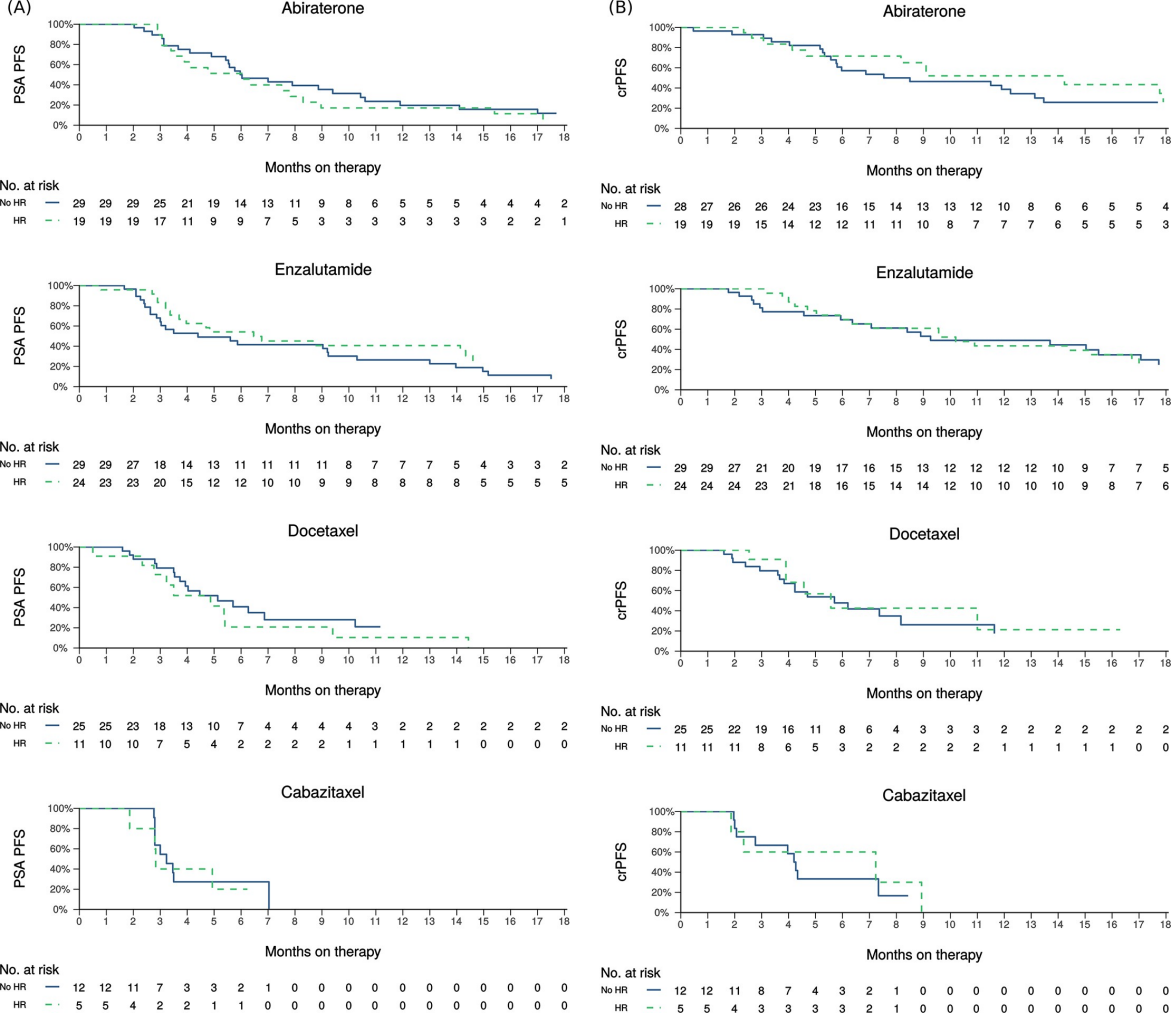
Kaplan-Meier curves of (A) PSA progression-free survival and (B) clinical or radiographic progression-free survival by treatment and HR status.

Kaplan-Meier curves of pPFS and crPFS are provided in Fig 2 by treatment modality. Median pPFS ranged between 2.8–6.5 months and median crPFS ranged between 4.2–14.2 months across treatment groups. There was no evidence that pPFS or crPFS differed based on HR repair gene mutation status for any treatment modality (Table 4).

**Table 4:** Median (A) PSA progression-free survival and (B) clinical or radiographic progression-free survival by treatment and HR status.

**A) PSA progression-free survival (pPFS)**				
**Treatment**	**HR Status**	**N**	**Median pPFS (95% CI)**	**P-value**
Abiraterone	No HR	29	6.0 (5.4, 10.6)	0.4
	HR	19	6.1 (3.9, 9.0)	
Enzalutamide	No HR	29	4.4 (3.0, 10.3)	0.15
	HR	24	6.5 (4.0, 24.0)	
Docetaxel	No HR	25	5.1 (3.7, NA)	0.2
	HR	11	4.9 (3.2, NA)	
Cabazitaxel	No HR	12	3.2 (2.8, NA)	0.7
	HR	5	2.8 (2.8, NA)	
**(B) Clinical or radiographic progression-free survival (crPFS)**				
**Treatment**	**HR Status**	**N**	**Median crPFS (95% CI)**	**P-value**
Abiraterone	No HR	28	8.0 (5.8, 13.5)	0.6
	HR	19	14.2 (8.2, NA)	
Enzalutamide	No HR	29	9.3 (6.4, 19.3)	0.5
	HR	24	10.2 (6.2, 19.5)	
Docetaxel	No HR	25	5.7 (4.2, NA)	0.7
	HR	11	5.6 (3.9, NA)	
Cabazitaxel	No HR	12	4.2 (2.8, NA)	0.6
	HR	5	7.2 (2.3, NA)	

P-values from log-rank tests. NA = not achieved.

Exploratory analyses comparing patients with mutations in *BRCA2* or *PALB2* to patients without HR repair mutations did not reveal differences in PSA30/PSA50 or pPFS/crPFS for any treatment (S1 and S2 Figs, S7 and S8 Tables), although the sample size within any molecular subgroup was small.

## Discussion

The principal impetus behind this study was to evaluate whether mCRPC patients harboring mutations both directly and indirectly involved in HR repair achieve comparable outcomes to patients without such mutations following treatment with conventional, regulatory-approved treatment regimens. Given the recent FDA approvals of the PARP inhibitors rucaparib and olaparib, as well as interest in developing platinum-based chemotherapy for patients with HR repair gene mutations, we felt it was imperative to examine whether current conventional mCRPC treatments retain efficacy in this patient population. Our findings of similar PSA response and PFS in patients with HR repair gene mutations treated with ARSIs and taxane-based chemotherapy suggests that current standard mCRPC treatments are still very reasonable options for this patient population.

Our data largely aligns with the results of the recently published PROREPAIR-B study, which found non-significant differences in PSA50, PFS, and cause-specific survival when comparing germline *ATM/BRCA1/BRCA2/PALB2* carriers to non-carriers [35]. In contrast to the PROREPAIR-B study, which reported decreased survival in men with *BRCA2* mutations in a post hoc analysis, we did not observe survival differences in those with *BRCA2* mutations; although, our small sample size limited our statistical power to detect differences in outcomes. It does seem plausible that *BRCA1/2* and other proteins directly interacting with the *BRCA* complex may have distinct clinical and biologic relevance compared to those that indirectly regulate HR repair (e.g., *CDK12*, *ATM*, *CHEK2*). Indeed, results from TRITON2, TOPARP-B, and PROfound all suggest that responses to PARP inhibitors are largely observed in those with *BRCA* mutations [12, 13, 15]. These observations support the need to further examine clinical outcomes on an individual gene basis.

The 37% overall HR repair gene mutation rate found within our population is significantly higher than what has been reported in previous studies. It is important to emphasize that our research was not designed to characterize the frequency of HR repair gene mutations within mCRPC patients, but rather to assess clinical response in patients with such mutations. Our population was not cross-sectional and was enriched by our selection criteria. Specifically, our requirement of NGS likely inflated the frequency of HR repair gene mutations found, as NGS was more often performed in patients with significant family history, high-risk tumor histology (e.g., Gleason grade group 4–5 and ductal histology) [5, 36, 37], unique clinical course, or known germline variants.

A major limitation of this study was its small sample size. With 90 total patients and relatively small numbers in each treatment modality, our analyses were underpowered to detect small differences in outcomes. Limited patient numbers also precluded additional sub-analyses, including comparisons of germline versus somatic mutations, monoallelic versus biallelic mutations, and *BRCA1/2*-mutated versus other HR repair gene alterations. We also used a permissive approach for classifying HR repair deficiency. This approach was largely pragmatic in nature and resembles the ‘real world’ data most practicing oncologists use to make treatment choices given that many NGS platforms do not report LOH events or germline alterations. Until such data becomes more readily reported and operational for providers, our data indicates that standard therapies for mCRPC (i.e. non-DDR-targeted treatments such as ARSIs and taxane chemotherapy) should still be considered for these patients.

The retrospective nature of this study provided its own set of challenges. NGS was performed at variable times during clinical courses, so HR repair gene mutation status may have been unknown at the time of treatment in many patients. While some data suggests that HR repair gene mutations are typically early (i.e. truncal) genomic events, we cannot rule out the possibility that some patient had their HR gene mutational status misclassified [38]. Additionally, when assessing PFS endpoints, we did not require confirmation of PSA or clinical/radiographic progression. No central review was performed, and there were no preset criteria for the evaluation of progression events. PFS outcomes were also dependent on the intervals at which clinical markers for progression were assessed. Whereas a prospective trial could standardize the frequency of clinical evaluations across all patients, ours were entirely provider dependent.

Another potential constraint was our exclusion of treatments lasting less than 60 days. Consistent with PCWG3 guidelines, our intent was to only evaluate patients who had received a sufficient duration of treatment to determine if they would achieve any therapeutic response. In this regard, patients who may have been taken off therapy prematurely for rising PSA values or symptoms consistent with clinical progression would not obscure the remainder of the data set. However, if there happened to be an association between HR repair gene status and frequency of early-onset progression events, it could be indicative of a bias that was manufactured through our inclusion criteria.

Future directions should include an effort to more definitively describe treatment responses in mCRPC patients with HR repair gene mutations using larger, prospective trials with standardized clinical outcomes. In addition, more granular outcomes data for each specific HR repair gene are needed to determine which are predictive and prognostic biomarkers.

## Conclusion

In our retrospective analysis of patients with mCRPC, HR repair gene mutational status associated with similar PSA response and PFS following treatment with ARSIs and taxane-based chemotherapy. Available data suggests that standard therapies should still be considered for patients with HR repair gene mutations. Additional prospective and larger studies are needed to confirm these findings.

## Supporting information

S1 Fig Waterfall plot of best PSA response by treatment and HR status (BRCA2 or PALB2 vs no HR). Maximum percent relative change from baseline during treatment or after completing treatment but prior to subsequent treatment. Maximum percent change greater than 100% is truncated at 100%. Dashed horizontal lines show 50% decrease.
(TIF)

S2 Fig Kaplan-Meier curves of (A) PSA progression-free survival and (B) clinical or radiographic progression-free survival by treatment and HR status (BRCA2 or PALB2 vs no HR).
(TIFF)

S1 Table Specific genetic variants of every patient included in the HR cohort. “WEC” denotes the Whole Exome Sequencing assay.
(PDF)

S2 Table Baseline lab comparisons at start of abiraterone based on HR status. P-values for continuous measures from Kruskal-Wallis rank sum test and for categorical measures from Fisher’s exact test.
(PDF)

S3 Table Baseline lab comparisons at start of enzalutamide based on HR status. P-values for continuous measures from Kruskal-Wallis rank sum test and for categorical measures from Fisher’s exact test.
(PDF)

S4 Table Baseline lab comparisons at start of docetaxel based on HR status. P-values for continuous measures from Kruskal-Wallis rank sum test and for categorical measures from Fisher’s exact test.
(PDF)

S5 Table Baseline lab comparisons at start of cabazitaxel based on HR status. P-values for continuous measures from Kruskal-Wallis test rank sum and for categorical measures from Fisher’s exact test.
(PDF)

S6 Table Predicted probabilities of (A) PSA30 and (B) PSA50 adjusted for prior treatment with similar therapy.
(PDF)

S7 Table Best PSA response by treatment and HR status (BRCA2 or PALB2 vs no HR). PSA50 is 50% decrease in PSA relative to baseline. PSA30 is 30% decrease in PSA relative to baseline. P-values from Fisher’s exact test.
(PDF)

S8 Table Median (A) PSA progression-free survival and (B) clinical or radiographic progression-free survival by treatment and HR status (BRCA2 or PALB2 vs no HR). P-values from log-rank tests. NA = not achieved.
(PDF)
