# Supplementary figures and images for: Impact of mutations in homologous recombination repair genes on treatment outcomes for metastatic castration resistant prostate cancer
Source: PLoS One. 2020 Sep 30;15(9):e0239686. doi: 10.1371/journal.pone.0239686 (PMC7526881; doi:10.1371/journal.pone.0239686)

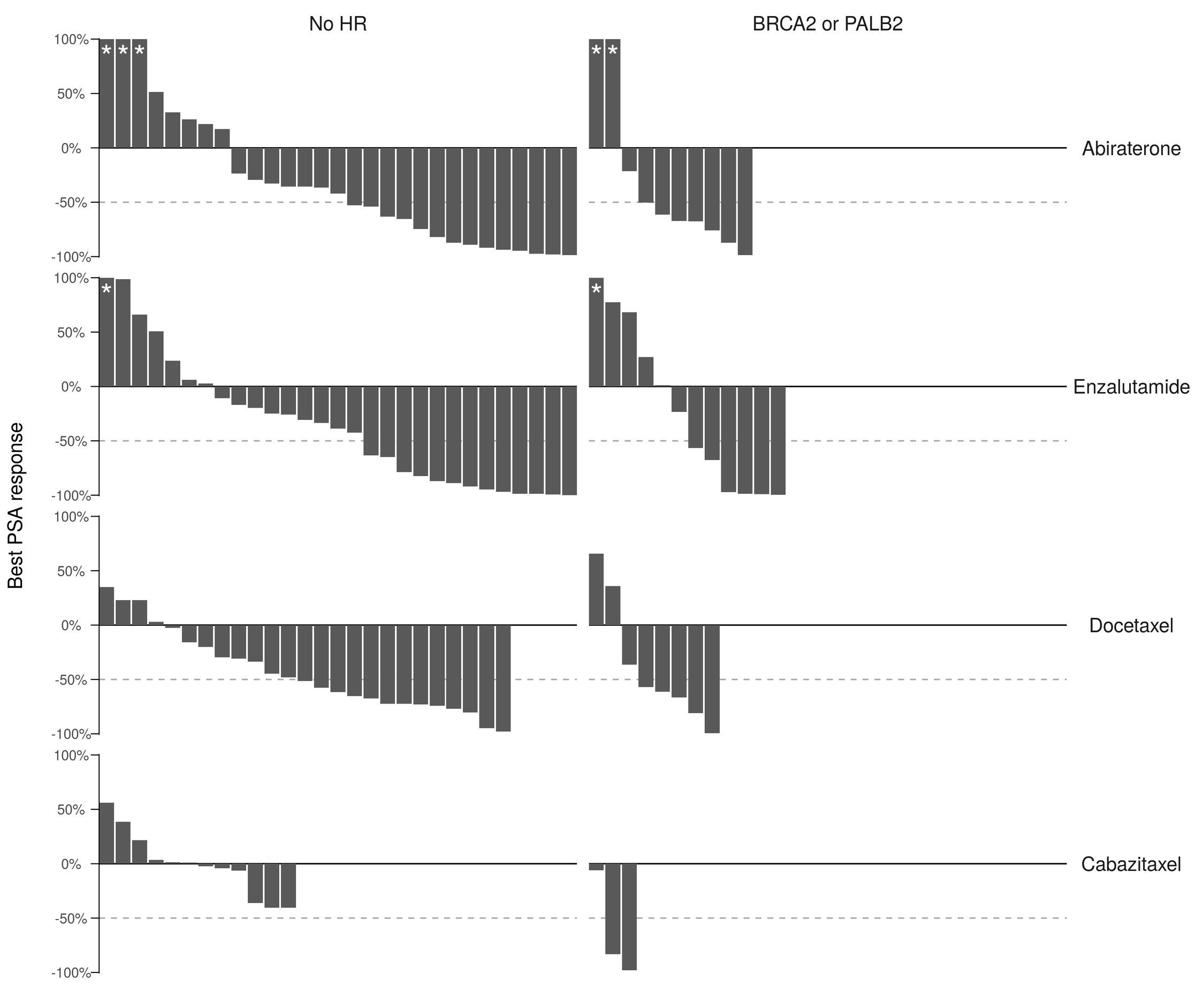

Supplement: S1 Fig — Maximum percent relative change from baseline during treatment or after completing treatment but prior to subsequent treatment. Maximum percent change greater than 100% is truncated at 100%. Dashed horizontal lines show 50% decrease. (TIF) [file pone.0239686.s001.tif]

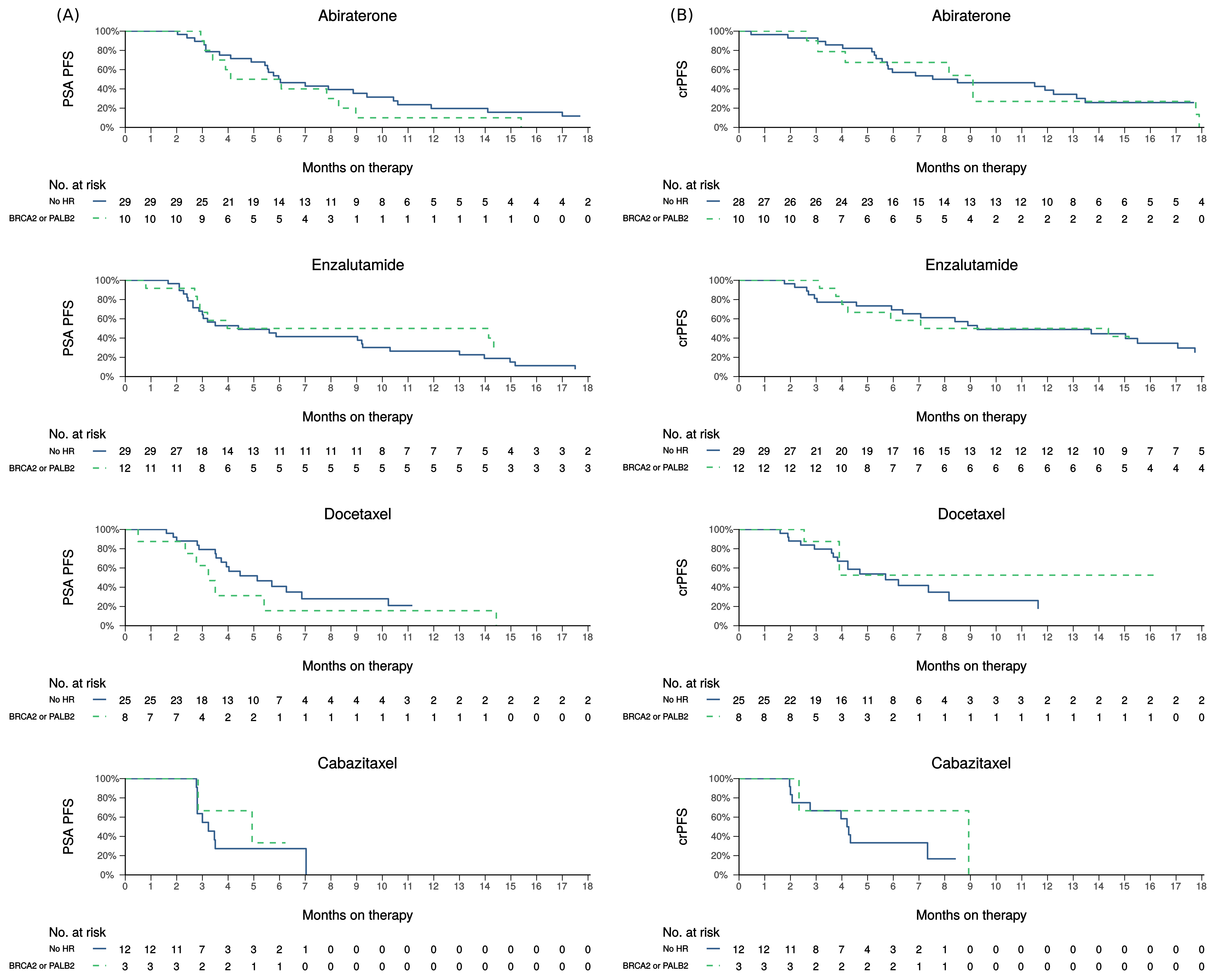

Supplement: S2 Fig — Kaplan-Meier curves of (A) PSA progression-free survival and (B) clinical or radiographic progression-free survival by treatment and HR status (BRCA2 or PALB2 vs no HR). (TIFF) [file pone.0239686.s002.tiff]
