# Supplementary material for: Impact of mutations in homologous recombination repair genes on treatment outcomes for metastatic castration resistant prostate cancer
Source: PLoS One. 2020 Sep 30;15(9):e0239686. doi: 10.1371/journal.pone.0239686 (PMC7526881; doi:10.1371/journal.pone.0239686)
Supplement: S1 Table — “WEC” denotes the Whole Exome Sequencing assay. (PDF) [file pone.0239686.s003.pdf]

S1 Table. Specific genetic variants of every patient included in the HR cohort.

Study ID	Sequencing Assay	Tissue Type (1=primary, 2=metastatic, 3=ctDNA, 4=germline)	Affected HR Gene	Variant type	Germline alteration detected?	Bi-allelic mutation detected?	Notes
1	UW-OncoPlex	2	FANCA	Copy loss on portions of chromosome 16	N/A	No	
2	WEC	1	BRCA2	Copy loss	No	Yes	Germline testing as part of WEC was negative.
4	UW-OncoPlex	3	BRCA2	chr13:g.32920928-32920952del, NM_000059.3:c.6938-12_6938-36del	N/A	Yes	Patient had a 23bp somatic deletion mutation in BRCA2 within intron 12 near the exon 13 splice boundary in BRCA2 gene. The sample also tested positive for a VUS in CDK12 (p.D877Y,NM_015083.2:c.2629 G>T).
9	UW-OncoPlex	1	MRE11A	p.E506X, NM_005590.3:c.1516 G to T	N/A	No	
14	UW-OncoPlex	2	BRCA2	Copy loss	No	Yes	Patient also had negative germline testing with BROCA.
20	UW-OncoPlex	3	BRCA2	Copy loss	No	Yes	The patient had two ctDNA samples tested a year apart. The first sample was collected prior to the initiation of olaparib treatment. Both samples were positive for BRCA2 copy loss, although the loss was much less pronounced in the second study, suggesting the possibility of selection of a BRCA2-intact tumor clone under treatment selective pressure.
24	FoundationOne, Color	1, 4	BRCA2	C.7558C>T	Yes	Yes	Germline testing with Color was positive for BRCA2 C.7558C>T mutation (this is also called p.Arg2520*, which is a known truncating pathogenic variant). Somatic testing with FoundationOne also showed Arg2520* mutation in BRCA2.
25	UW-OncoPlex	1	CDK12	NM_015083:c.2963+1 G to T, p.H194Kfs*133, NM_015083.1:c.576_609del	N/A	Yes	
27	UW-OncoPlex	2	BRCA2	Copy loss on a portion of chromosome 13	N/A	No	
28	UW-OncoPlex, WEC	1	BRCA2	p.V2969Cfs*7, NM_000059.3:c.8904delC	Yes	Yes	Patient had known germline BRCA2 mutation through WEC testing, confirmed to have somatic BRCA2 mutation through UW-OncoPlex testing.

31	UW-OncoPlex	1	BRCA2	Copy loss	N/A	Yes	
33	UW-OncoPlex	2	BRCA2	Copy loss on a portion of chromosome 13	N/A	No	
34	UW-OncoPlex	3	CDK12	p.E928Nfs*18, NM_015083.2:c.2778del, NM_015083.2:c.1046 G to T and NM_015083.2:c.1046+6 C to G	N/A	Yes	
36	FoundationOne	1	ATM	p.R3047*	N/A	Yes	
38	UW-OncoPlex	2	PALB2	p.L345Sfs*2, NM_024675.3:c.1032_1033dup	N/A	Yes	
39	UW-OncoPlex	1	CHD1	p.K1352Vfs*18, NM_001270.2:c.4054_4057del	N/A	No	
43	UW-OncoPlex	1	BRCA2	Copy loss of a portion of chromosome 13	N/A	N/A	
44	Color	4	BRCA2	N/A	Yes	N/A	Information on variant was not available in patient records. This mutation was included because of the high penetrance and frequency of a second hit associated with BRCA2 mutations. Additionally, it enabled the patient to enroll in a PARP inhibitor trial.
48	UW-OncoPlex	1	CHD1	Copy loss	N/A	Yes	
57	FoundationOne	1	CDK12	E647fs*8, R100fs*27	N/A	Yes	
59	UW-OncoPlex	1	BRCA2	p.Y1762X, NM_000059.3:c.5286T>G	N/A	Yes	The tissue also tested positive for rare variant in CDK12 (p.G923V, NM_015083.2:c.2768G>T) thought to represent benign variation.
63	BRACAnalysis	4	BRCA2	5057delTG	Yes	N/A	Patient did not have sequencing of tumor tissue. This mutation was included because of the high penetrance and frequency of a second hit associated with BRCA2 mutations.
69	UW-OncoPlex	1	CHD1	Copy loss	N/A	Yes	
70	UW-OncoPlex	2	ATM	p.E2847_L2850del, NM_000051.3:c.8540_8551del	N/A	No	The ATM mutation occurred within the critical PI3K domain, in which other mutations have been shown to be pathogenic. The sample also tested positive for copy losses on portions of chromosomes 11, 12, 13 (including single copy loss of BRCA2), 16 (including single copy loss of FANCA), 17 (including single copy loss of BRCA1).

71	UW-OncoPlex	1	FANCA	N/A	N/A	N/A	Information on variant was not available in patient records. This mutation was included because it prompted treatment with Carboplatin due to the association between FANCA mutations and sensitivity to platinum chemotherapy.
72	UW-OncoPlex	1	CDK12	p.Y1329Cfs*4, NM_015083.2:c.3986_3991delinsGTGG	N/A	N/A	
78	Guardant360, FoundationOne, Color	2, 3, 4	BRCA2	p.T2125fs*12	Yes	N/A	Patient had ctDNA testing with Guardant360, metastatic tumor testing with FoundationOne, and germline testing with Color that all confirmed the same frameshift mutation.
79	UW-OncoPlex	3	CHD1	Copy loss	N/A	Yes	
81	FoundationOne	1	ATM	Loss of exons 9-52	No	No	Patient also had testing germline testing with Color that revealed VUS for MSH6 (c.33C>G phe11leu).
82	GeneTrails	1	FANCA	Copy loss	N/A	N/A	The sample also tested positive for a VUS in ATR (p.I2164M)
84	UW-OncoPlex	1	BRCA2	p.Q2530X, NM_000059.3:c.7588 C to T	N/A	Yes	This mutation results in a premature stop codon at position 2530 in exon 15 of the BRCA2 protein.
86	UW-OncoPlex	2	MRE11A	p.L56W, NM_005590.3:c.167 T to G, p.K633Tfs*45, NM_005590.3:c.1876_1895dup	N/A	Yes	
90	UW-OncoPlex	1	BRCA2, CHEK2	BRCA2: p.Q3066X, NM_000059.3:c.9196C to T, chr13:32912206 to 32912334 del;<br><br>CHEK2: p.(T367Mfs*15), NM_007194.3:c.1100delC	N/A	Yes	BRCA2: The first mutation results in a premature stop codon and has been previously described as an inherited mutation. The second deleterious BRCA2 mutation is an approximately 127bp deletion within exon 11 of the BRCA2 gene.
