# Supplementary material for: Impact of mutations in homologous recombination repair genes on treatment outcomes for metastatic castration resistant prostate cancer
Source: PLoS One. 2020 Sep 30;15(9):e0239686. doi: 10.1371/journal.pone.0239686 (PMC7526881; doi:10.1371/journal.pone.0239686)
Supplement: S2 Table — P-values for continuous measures from Kruskal-Wallis rank sum test and for categorical measures from Fisher’s exact test. (PDF) [file pone.0239686.s004.pdf]

S2 Table. Baseline lab comparisons at start of abiraterone based on HR status

Measure	No HR (N=29)	HR (N=19)	P-value
Albumin, median [IQR]	4.00 [3.80, 4.20]	3.90 [3.68, 4.23]	0.6
Alk Phos, median [IQR]	104.00 [65.00, 201.00]	91.00 [75.50, 115.50]	0.4
Hemoglobin, median [IQR]	12.80 [11.60, 13.78]	12.10 [11.59, 13.30]	0.6
LDH, median [IQR]	165.00 [156.50, 173.75]	140.00 [135.50, 208.50]	0.9
Neutrophil, median [IQR]	4.21 [2.91, 4.58]	4.35 [2.44, 5.84]	0.5
Platelets, median [IQR]	205.00 [178.00, 252.00]	215.00 [182.50, 242.50]	0.9
Testosterone, median [IQR]	0.40 [0.10, 1.00]	0.20 [0.05, 0.40]	0.3
WBC, median [IQR]	6.45 [5.41, 7.96]	6.32 [4.62, 7.87]	1.0
ECOG, N (%)			
- 0	16 (55.2)	11 (57.9)	
- 1	10 (34.5)	7 (36.8)	1.0
- Unknown	3 (10.3)	1 (5.3)	
Prior abiraterone or enzalutamide, N (%)	4 (13.8)	2 (10.5)	1.0
