# Supplementary material for: Impact of mutations in homologous recombination repair genes on treatment outcomes for metastatic castration resistant prostate cancer
Source: PLoS One. 2020 Sep 30;15(9):e0239686. doi: 10.1371/journal.pone.0239686 (PMC7526881; doi:10.1371/journal.pone.0239686)
Supplement: S3 Table — P-values for continuous measures from Kruskal-Wallis rank sum test and for categorical measures from Fisher’s exact test. (PDF) [file pone.0239686.s005.pdf]

S3 Table. Baseline lab comparisons at start of enzalutamide based on HR status.

Measure	No HR (N=29)	HR (N=24)	P-value
Albumin, median [IQR]	4.00 [3.80, 4.20]	3.90 [3.68, 4.23]	0.6
Alk Phos, median [IQR]	80.00 [70.50, 122.50]	82.00 [65.00, 110.50]	0.7
Hemoglobin, median [IQR]	12.00 [11.15, 13.35]	12.55 [12.10, 13.92]	0.3
LDH, median [IQR]	155.00 [144.50, 170.50]	159.00 [145.00, 169.00]	0.9
Neutrophil, median [IQR]	.33 [2.69, 5.00]	3.05 [2.65, 3.83]	0.5
Platelets, median [IQR]	195.00 [165.50, 217.50]	208.50 [174.50, 262.50]	0.5
Testosterone, median [IQR]	0.08 [0.05, 0.30]	0.20 [0.09, 0.20]	1.0
WBC, median [IQR]	5.20 [4.62, 7.32]	5.33 [4.73, 6.29]	0.9
ECOG, N (%)			
- 0	14 (48.3)	13 (54.2)	
- 1	7 (24.1)	5 (20.8)	
- 2	2 (6.9)	4 (16.7)	0.5
- 3	3 (10.3)	0 (0.0)	
- Unknown	3 (10.3)	2 (8.3)	
Prior abiraterone or enzalutamide, N (%)	17 (58.6)	11 (45.8)	0.4
