# Supplementary material for: Impact of mutations in homologous recombination repair genes on treatment outcomes for metastatic castration resistant prostate cancer
Source: PLoS One. 2020 Sep 30;15(9):e0239686. doi: 10.1371/journal.pone.0239686 (PMC7526881; doi:10.1371/journal.pone.0239686)
Supplement: S4 Table — P-values for continuous measures from Kruskal-Wallis rank sum test and for categorical measures from Fisher’s exact test. (PDF) [file pone.0239686.s006.pdf]

S4 Table. Baseline lab comparisons at start of docetaxel based on HR status.

Measure	No HR (N=25)	HR (N=11)	P-value
Albumin, median [IQR]	4.00 [3.75, 4.20]	3.95 [3.73, 4.15]	0.8
Alk Phos, median [IQR]	101.50 [64.00, 253.25]	89.50 [65.75, 93.75]	0.3
Hemoglobin, median [IQR]	12.20 [10.07, 12.95]	13.10 [10.90, 13.55]	0.3
LDH, median [IQR]	228.00 [215.00, 364.00]	203.00 [177.50, 228.50]	0.5
Neutrophil, median [IQR]	3.84 [3.04, 5.71]	4.10 [2.51, 5.33]	0.7
Platelets, median [IQR]	243.50 [167.25, 276.00]	220.00 [200.00, 266.50]	0.8
Testosterone, median [IQR]	0.05 [0.05, 0.09]	N/A	N/A
WBC, median [IQR]	5.48 [4.21, 7.10]	6.40 [4.98, 7.07]	0.4
ECOG, N (%)			
- 0	5 (20.0)	5 (45.5)	
- 1	14 (56.0)	2 (18.2)	
- 2	4 (16.0)	2 (18.2)	0.17
- 3	1 (4.0)	1 (9.1)	
- Unknown	1 (4.0)	1 (9.1)	
Prior docetaxel or cabazitaxel, N (%)	2 (8.0)	0 (0.0)	1.0
