# Supplementary material for: Impact of mutations in homologous recombination repair genes on treatment outcomes for metastatic castration resistant prostate cancer
Source: PLoS One. 2020 Sep 30;15(9):e0239686. doi: 10.1371/journal.pone.0239686 (PMC7526881; doi:10.1371/journal.pone.0239686)
Supplement: S5 Table — P-values for continuous measures from Kruskal-Wallis test rank sum and for categorical measures from Fisher’s exact test. (PDF) [file pone.0239686.s007.pdf]

S5 Table. Baseline lab comparisons at start of cabazitaxel based on HR status.

Measure	No HR (N=12)	HR (N=5)	P-value
Albumin, median [IQR]	3.80 [3.77, 3.92]	3.30 [3.20, 3.40]	0.09
Alk Phos, median [IQR]	69.00 [61.75, 96.50]	86.00 [79.00, 125.00]	0.4
Hemoglobin, median [IQR]	11.20 [10.45, 11.93]	12.00 [9.90, 12.60]	0.6
LDH, median [IQR]	217.50 [213.25, 221.75]	263.00 [234.50, 516.50]	0.6
Neutrophil, median [IQR]	4.50 [3.73, 6.27]	3.54 [2.45, 4.67]	0.2
Platelets, median [IQR]	200.50 [165.75, 240.25]	88.00 [79.00, 109.00]	0.03
Testosterone, median [IQR]	0.05 [0.05, 0.05]	0.05 [0.05, 0.05]	N/A
WBC, median [IQR]	5.95 [4.63, 8.19]	6.03 [3.80, 6.19]	0.5
ECOG, N (%)			
- 0	1 (8.3)	1 (20.0)	0.4
- 1	9 (75.0)	2 (40.0)	
- 2	2 (16.7)	2 (40.0)	
Prior docetaxel or cabazitaxel, N (%)	11 (91.7)	5 (100.0)	1.0
