# Supplementary material for: Impact of mutations in homologous recombination repair genes on treatment outcomes for metastatic castration resistant prostate cancer
Source: PLoS One. 2020 Sep 30;15(9):e0239686. doi: 10.1371/journal.pone.0239686 (PMC7526881; doi:10.1371/journal.pone.0239686)
Supplement: S6 Table — Predicted probabilities of (A) PSA30 and (B) PSA50 adjusted for prior treatment with similar therapy. (PDF) [file pone.0239686.s008.pdf]

S6 Table. Predicted probabilities of (A) PSA30 and (B) PSA50 adjusted for prior treatment with similar therapy.

(A) PSA30

Treatment	Prior therapy	N	No HR	HR
Abiraterone	No	42	73%	79%
	Yes	6	21%	27%
Enzalutamide	No	25	89%	92%
	Yes	28	38%	45%
Docetaxel	No	34	67%	72%
	Yes	2	88%	N/A
Cabazitaxel	No	1	69%	N/A
	Yes	16	23%	37%

(B) PSA50

Treatment	Prior therapy	N	No HR	HR
Abiraterone	No	42	54%	72%
	Yes	6	18%	31%
Enzalutamide	No	25	86%	95%
	Yes	28	18%	40%
Docetaxel	No	34	50%	62%
	Yes	2	85%	N/A
Cabazitaxel	No	1	3%	N/A
	Yes	16	3%	34%
