# Supplementary material for: Impact of mutations in homologous recombination repair genes on treatment outcomes for metastatic castration resistant prostate cancer
Source: PLoS One. 2020 Sep 30;15(9):e0239686. doi: 10.1371/journal.pone.0239686 (PMC7526881; doi:10.1371/journal.pone.0239686)
Supplement: S7 Table — PSA50 is 50% decrease in PSA relative to baseline. PSA30 is 30% decrease in PSA relative to baseline. P-values from Fisher’s exact test. (PDF) [file pone.0239686.s009.pdf]

S7 Table. Best PSA response by treatment and HR status (BRCA2 or PALB2 vs no HR).

Treatment	Response	No HR, N (%)	BRCA2 or PALB2, N (%)	P-value
Abiraterone	PSA30	19/29 (66%)	7/10 (70%)	1.0
	PSA50	14/29 (48%)	7/10 (70%)	0.3
Enzalutamide	PSA30	17/29 (59%)	6/12 (50%)	0.7
	PSA50	13/29 (45%)	6/12 (50%)	1.0
Docetaxel	PSA30	17/25 (68%)	6/8 (75%)	1.0
	PSA50	13/25 (52%)	5/8 (62%)	0.7
Cabazitaxel	PSA30	3/12 (25%)	2/3 (67%)	0.2
	PSA50	0/12 (0%)	2/3 (67%)	0.03
