# Supplementary material for: Impact of mutations in homologous recombination repair genes on treatment outcomes for metastatic castration resistant prostate cancer
Source: PLoS One. 2020 Sep 30;15(9):e0239686. doi: 10.1371/journal.pone.0239686 (PMC7526881; doi:10.1371/journal.pone.0239686)
Supplement: S8 Table — Median (A) PSA progression-free survival and (B) clinical or radiographic progression-free survival by treatment and HR status (BRCA2 or PALB2 vs no HR). P-values from log-rank tests. NA = not achieved. (PDF) [file pone.0239686.s010.pdf]

S8 Table. Median (A) PSA progression-free survival and (B) clinical or radiographic progression-free survival by treatment and HR status (BRCA2 or PALB2 vs no HR).

(A) PSA progression-free survival (pPFS)

Treatment	HR Status	N	Median pPFS (95% CI)	P-value
Abiraterone	No HR	29	6.0 (5.4, 10.6)	0.3
	BRCA2 or PALB2	10	5.1 (3.4, NA)	
Enzalutamide	No HR	29	4.4 (3.0, 10.3)	0.2
	BRCA2 or PALB2	12	9.1 (2.9, NA)	
Docetaxel	No HR	25	5.1 (3.7, NA)	0.12
	BRCA2 or PALB2	8	3.2 (2.8, NA)	
Cabazitaxel	No HR	12	3.2 (2.8, NA)	0.6
	BRCA2 or PALB2	3	4.9 (2.8, NA)	

(B) Clinical or radiographic progression-free survival (crPFS)

Treatment	HR Status	N	Median crPFS (95% CI)	P-value
Abiraterone	No HR	28	8.0 (5.8, 13.5)	0.5
	BRCA2 or PALB2	10	9.1 (4.1, NA)	
Enzalutamide	No HR	29	9.3 (6.4, 19.3)	0.7
	BRCA2 or PALB2	12	10.7 (4.2, NA)	
Docetaxel	No HR	25	5.7 (4.2, NA)	0.5
	BRCA2 or PALB2	8	NA (3.9, NA)	
Cabazitaxel	No HR	12	4.2 (2.8, NA)	0.3
	BRCA2 or PALB2	3	8.9 (2.3, NA)	
